# Anticipatory Cortisol Regulation During Competition in Elite Female Field Hockey Players

**DOI:** 10.1002/smi.70199

**Published:** 2026-07-05

**Authors:** Jorge García‐Bastida, Manuel Jiménez, Jana Gallardo‐Pérez, Iván Rivilla‐Arias, Jerónimo Carmelo García‐Romero, Guillermo Sánchez‐Martínez

**Affiliations:** ^1^ Facultad de Educación y Humanidades Universidad Internacional de la Rioja Logroño Spain; ^2^ Facultad de Medicina Universidad de Málaga Málaga Spain

**Keywords:** competitive anxiety, cortisol awakening response, elite female field hockey, outcome, psychophysiological response to competition, stress

## Abstract

Research examining the cortisol awakening response (CAR) in elite female athletes during official competition remains limited, particularly under ecologically valid high‐stakes conditions. This study investigated associations between pre‐competitive Delta CAR, competitive outcome, anxiety‐related variables, and cortisol fluctuations on an official competition day among elite female field hockey players. Fifty‐two elite female field hockey players participated in this study during the Spanish Championship Cup. Salivary cortisol samples were collected at awakening (C0), 30 min after awakening (C1), 30 min before warm‐up (C2), and 30 min after competition (C3). CAR was calculated as the difference between C1 and C0. Athletes also completed the Self‐Assessment Manikin (SAM), Likert‐style affective self‐reports, and the Competitive State Anxiety Inventory‐2 Revised (CSAI‐2). Significant within‐subject cortisol fluctuations were observed across the competition day. Players who eventually lost their matches exhibited higher CAR values than those who eventually won (rank‐biserial *r* = 0.58, *p* < 0.001). CAR was moderately associated with cognitive anxiety, somatic anxiety, perceived difficulty, and arousal‐related variables. In addition, defeated players showed higher post‐competition cortisol concentrations than winners. In an ecologically valid elite competition setting, CAR varied substantially between athletes, showed partial associations with anxiety‐related and affective variables, and was associated with eventual competitive outcome. These findings suggest that CAR may reflect a context‐sensitive anticipatory endocrine response associated with competitive demands rather than a deterministic biomarker of stress or performance.

## 1 Introduction

Psychological and physiological responses play an important role in competitive performance in elite sport. Athletes competing at high levels are repeatedly exposed to substantial physical, cognitive, and emotional demands, including psychological pressure, fatigue, rapid decision‐making, uncertainty, and performance‐related stress (Jiménez et al. 2012; Kallen et al. 2017; Jiménez et al. 2020, 2026). In this context, activation of the hypothalamic–pituitary–adrenal (HPA) axis contributes to the regulation of physiological responses to competitive demands through glucocorticoid secretion, particularly cortisol, which mobilises energy resources and supports adaptation to stressful situations (Dickerson and Kemeny 2004; Lupien et al. 2009). Field hockey is a fast‐paced team sport characterised by repeated high‐intensity efforts, rapid perceptual processing, tactical decision‐making, and sustained attentional demands, all of which may contribute to psychophysiological stress during official competition (Morris‐Binelli et al. 2020; Malcolm et al. 2022).

The cortisol awakening response (CAR) is a circadian phenomenon characterised by a rapid increase in cortisol concentrations during the first 30–45 min after awakening, followed by a progressive decline throughout the day (Stalder et al. 2016). CAR has been widely studied as an indicator of HPA‐axis activity and has been associated with anticipation of forthcoming demands, workload, psychosocial stress, and cognitive‐emotional preparation (Clow et al. 2010; Fries et al. 2009). According to the anticipation hypothesis, CAR has been interpreted as a physiological response to anticipated daily challenges (Fries et al. 2009). Elevated CAR magnitudes have been reported in situations involving increased cognitive, emotional, or social‐evaluative demands, including demanding workdays, academic challenges, and competitive environments (Brant et al. 2010; Elder et al. 2018; Kunz‐Ebrecht et al. 2004; Wetherell et al. 2015). Previous research in female athletes has suggested that hormonal fluctuations during the menstrual cycle may partially influence cortisol awakening dynamics and autonomic regulation. These findings highlight the importance of considering sex‐specific neuroendocrine factors when interpreting CAR responses in elite sportswomen (Kayacan et al. 2021).

In sport settings, previous research has shown that cortisol concentrations frequently increase before competition across different disciplines, including tennis, motorcycling, dance, soccer, and swimming (Aizawa et al. 2006; Filaire et al. 2007, 2009; Meggs et al. 2016; Rohleder et al. 2007). However, findings regarding the relationship between CAR and competitive outcome. Meggs et al. (2016), for example, observed higher CAR values in swimmers who won their events, whereas Filaire et al. (2001) reported no significant differences between winners and losers in judo competitors. Other studies have reported attenuated or absent competition‐related CAR responses in athletes exposed repeatedly to similar competitive demands (MacDonald and Wetherell 2019; Strahler et al. 2010). These inconsistencies may be related to differences in athlete level, competitive context, sampling procedures, sex‐related factors, or sport‐specific psychological and physiological demands. Recent evidence in national‐level female endurance athletes has also shown that competition‐related increases in cortisol awakening responses and psychophysiological stress markers may be associated with race difficulty and performance‐related outcomes under ecologically valid conditions (Aydemir et al. 2025).

Current consensus guidelines recommend assessing CAR using multiple samples during the first hour after awakening in order to characterise the full temporal dynamics of cortisol secretion (Stalder et al. 2016). Nevertheless, implementing these procedures in elite sport environments during official competition is often difficult due to logistical and ecological constraints. Previous work in elite athletes has shown that simplified two‐sample protocols (awakening and +30 min) are sufficiently sensitive to detect competition‐related changes in HPA‐axis activity under real‐world conditions (MacDonald and Wetherell 2019). Consequently, abbreviated CAR protocols have become increasingly common in applied sport psychophysiology research because they minimise disruption to athletes' routines while preserving ecological validity during training camps and official competitions (Aguilar et al. 2018; Crewther et al. 2025).

Accumulating evidence also suggests that delta‐based CAR indices and area‐under‐the‐curve (AUC)‐derived measures may reflect partially distinct aspects of HPA‐axis functioning. Whereas AUC indices, particularly AUC with respect to ground (AUCg), primarily quantify total cortisol secretion across the sampling period, delta CAR indices isolate the rapid increase occurring immediately after awakening, which appears to be especially sensitive to anticipatory endocrine activation and forthcoming demands (Clow et al. 2010; Fries et al. 2009). High‐resolution analyses indicate that the principal secretory surge of the CAR occurs within the first 20–30 min after awakening, with peak activity generally reached before 30 min, while later sampling points increasingly reflect post‐peak decline and recovery‐related dynamics (Jiménez et al. 2026). Large cohort studies further suggest that CAR magnitude is relatively independent from total daily cortisol output, supporting the interpretation that early‐morning reactivity and cumulative cortisol exposure represent partially dissociable physiological processes.

In the present study, CAR was operationalised as the delta change between awakening cortisol concentrations and cortisol measured 30 min later (C1 − C0). This approach was selected because the primary objective was to examine the initial rise in cortisol secretion associated with anticipatory psychophysiological preparation before competition. In ecologically valid elite sport settings, where minimising interference with athletes' pre‐competition routines is essential, the use of a 0–30 min delta CAR index may provide a practical and sufficiently sensitive approach for capturing early anticipatory HPA‐axis activation under real‐world conditions. This early phase has been associated with anticipatory psychophysiological activation prior to expected demands (Clow et al. 2010; Fries et al. 2009). At the same time, it is important to acknowledge that abbreviated delta CAR protocols do not characterise the complete temporal dynamics of cortisol secretion following awakening and should therefore be interpreted specifically as an index of the initial rapid increase in cortisol associated with anticipatory activation, rather than as a comprehensive measure of total morning cortisol output.

Beyond training load and physiological stress, CAR has increasingly been interpreted as a context‐sensitive anticipatory response associated with the appraisal of forthcoming demands rather than as a simple marker of emotional distress (Fries et al. 2009; Stalder et al. 2025). In athletes, CAR has been associated with psychophysiological responses to training, competition, and recovery processes (Anderson and Wideman 2017; Anderson et al. 2018). However, despite growing interest in anticipatory endocrine responses in sport, little is known about how CAR behaves in elite female athletes competing under ecologically valid conditions in official competition. This limitation is particularly relevant given the relative underrepresentation of female athletes in psychophysiological research on competition and the documented sex‐related differences in CAR regulation.

Therefore, the aim of this study was to examine whether CAR varied among elite female field hockey players competing during an official national championship and whether these variations were associated with anxiety‐related variables, affective states, and eventual competitive outcome. We hypothesised that CAR would show substantial inter‐individual variability and would be partially associated with psychological variables and match outcome under high‐level competitive conditions.

## 2 Materials and Methods

### 2.1 Participants

This study included 52 elite female field hockey players competing in the Spanish ‘Copa de la Reina’ national championship. Participants represented all playing positions (4 goalkeepers, 21 defenders, 14 midfielders, and 13 forwards). Eleven athletes had competed in the Olympic Games, and 26 had represented their national teams, reflecting the high level of competition in the sample. Participants had a mean ± SD age of 23.50 ± 3.83 years, competitive experience of 15.54 ± 4.52 years, body mass of 59.32 ± 5.44 kg, height of 1.65 ± 0.20 m, and body mass index (BMI) of 21.71 ± 1.96 kg/m^2^. Additional descriptive information is presented in Table S1.

### 2.2 Measures and Instruments

#### 2.2.1 Cortisol

Salivary cortisol was measured as an index of HPA‐axis activity during competition day. Immediately upon awakening on competition day and again 30 min later, participants provided two samples of unstimulated whole saliva (2–5 mL) using Salivettes devices with a cotton swab (Sarstedt, Germany). For this study, the selected CAR measure was the 0–30 min interval (*C*_0_ = awakening; *C*_1_ = 30 min after awakening), which is considered a feasible abbreviated CAR protocol according to Jiménez et al. (2026). To measure differences in C outcome‐dependent samples, 30 min before warm‐up (*C*_2_), and 30 min after finishing the match (*C*_3_).

The night before the sampling day, they were explicitly informed not to eat, drink, smoke, or brush their teeth for 30 min before the sampling time. Players provided 2–5 mL saliva specimens in saliva collection devices; the CAR was obtained by subtracting the first C measurement from the second sample. Once the players had handed in the samples, the expedition team manager asked them if they had followed the instructions correctly. All samples (frozen at −30°C 15 min after collection) were kept in the refrigerator of our laboratory until they were analysed by a commercial enzyme‐linked immunosorbent assay (ELISA) with a Diametra kit (Milan, Italy) on an automatic analyser (Triturus, Grifols, Barcelona). C intra‐assay and inter‐assay coefficients of variation were less than 12%. The lower limits of detection for C kits were 3.5 pg/mL and 0.05 ng/mL. To minimise inter‐day variability, all samples from the same participant were analysed in duplicate within the same assay batch. Random duplicates were re‐assayed for quality control; values differing by > 10% were re‐measured, and mean concentrations were used for statistical analyses.

#### 2.2.2 Affective States

Affective states were assessed immediately after the second cortisol sample (C1), prior to warm‐up. Participants completed a battery of validated psychometric instruments to assess affective valence, arousal, and dominance using the Self‐Assessment Manikin (SAM; Lang 1995). Psychometric properties are well established: SAM ratings showed a Cronbach's alpha coefficient (*r* ≈ = 0.94–0.97).

To assess pre‐competitive anxiety, the Competitive State Anxiety Inventory‐2 (CSAI‐2; Martens et al. 1990; Andrade Fernández et al. 2007) was administered. This 27‐item questionnaire uses a 4‐point Likert scale ranging from 1 (‘strongly disagree’) to 4 (‘strongly agree’). It includes three subscales—somatic anxiety, cognitive anxiety, and self‐confidence—with acceptable internal consistency in the current sample (Cronbach's *α* = 0.72, 0.70, and 0.70, respectively) and adequate reproducibility (ICC = 0.89).

All questionnaires were administered in a quiet room, immediately after saliva collection, under the supervision of the research team to ensure compliance and standardisation. The combination of these instruments provided complementary information on athletes' emotional activation, affective tone, and competitive anxiety, offering complementary information on affective and anxiety‐related states.

### 2.3 Procedure

The study was approved by the Committee of Ethics with code: 35–2018‐H. Before the championship, meetings were conducted with club representatives to explain the study procedures in detail. Medical reports about physiological disorders, drug abuse, or psychiatric conditions were complemented. Informed consent was signed by each participant and each club. No participant indicated contraceptive use. The analysis was conducted in the Spanish ‘Copa de la Reina’ championships, where teams faced each other in single‐match playoffs. Players were selected for two matches based on the previous draw. These players belonged to the first four teams in the classification of the Honour A division of the Spanish National Hockey League. The first match pitted two teams from the city of Madrid (Madrid), Club de Campo Villa de Madrid (CDC) and San Pablo Valdeluz (SPV), while the other match pitted a team from the city of Terrasa (Catalonia), Club Egara (CE) and another from Sant Cugat (Catalonia), Junior Football Club (JFC). In addition, all these teams had been finalists or champions (or medallists) in previous leagues or Spanish ‘Copa de la Reina’ championships. Therefore, all participants were striving to reach the semi‐finals.

All matches were qualification for the semi‐finals, thereby ensuring a highly competitive context with strong ecological validity. The matches had four quarters of 17.5 min each, with a 5‐min rest period between the first and second halves and the third and fourth quarters. The halftime break (between the second and third quarters) lasted 15 min. The first match, which resulted in a 2‐0 JFC victory over EC (1‐0 at half‐time), started at noon, ended at 13:45; the second match, which resulted in a 1‐2 CDC victory over SPV (1‐1 at half‐time), started at 11:00, ended at 13:47, both matches taking place on 17 March.

### 2.4 Data Analyses

All statistical analyses were performed using SPSS v22 (IBM, New York, USA). Cortisol concentrations measured across the competition day (C0: awakening; C1: +30 min after awakening; C2: pre‐competition; C3: post‐competition) were analysed using a two‐way repeated‐measures ANOVA. Normality of hormonal variables was examined using the Kolmogorov–Smirnov test. Given the non‐normal distribution of cortisol levels, the data were logarithmically transformed before performing parametric analyses. Homogeneity of variances was assessed using Levene's test, and sphericity was evaluated using Mauchly's test; Greenhouse–Geisser corrections were applied when necessary. The repeated‐measures ANOVA included Time (C0, C1, C2, C3) as the within‐subjects factor and Match Outcome (i.e., Win vs. Loss) as the between‐subjects factor, allowing examination of the main effects of time and outcome and their interaction. Effect sizes were reported as partial eta squared (*η* ^2^_*p*_) or generalised eta squared (ges), as appropriate. To assess variation in cortisol concentration at the four time points (C0–C3), intraclass correlation coefficients (ICC; bidirectional mixed‐effects model) were used to quantify the relative contributions of the components both between and within subjects. Effect size was estimated using the rank‐biserial correlation (*r*) and its corresponding 95% confidence interval. CAR was calculated as the difference between cortisol concentrations measured 30 min after awakening (C1) and at awakening (C0), as described by Aguilar et al. (2018) and Crewther et al. (2025); and percentage changes in cortisol from pre‐to post‐competition were computed according to Jiménez et al. (2012), Jiménez et al. (2020) using the formula [(post − pre)/pre] × 100.

Differences in cortisol concentrations according to playing position (goalkeeper, defender, midfielder, forward) at individual sampling time points (C0–C3) were examined using one‐way ANOVA models on log‐transformed cortisol values. Given the exploratory nature of the correlational analyses, Bonferroni‐adjusted post‐hoc comparisons were performed. Emphasis was placed on the magnitude of effect sizes and the consistency of associations rather than on isolated *p*‐values. Associations between hormonal measures, pre‐competitive anxiety (CSAI‐2R), and affective states (SAM) were analysed using Spearman's rank correlation coefficient (rho). Statistical significance was set at *p* < 0.05. All data were screened prior to analysis. Observations with missing, incomplete, or methodologically invalid data were excluded. To reduce inflation of Type I error, only correlations with consistent effect sizes and theoretical coherence were interpreted, while the correlational analyses were considered exploratory. No missing‐data imputation was performed, and all analyses were conducted using only complete cases. A priori statistical power analysis was conducted using G*Power software (version 3.1). Assuming a medium effect size (*f* = 0.25), an alpha level of 0.05, four groups, and four repeated measurements, the estimated minimum sample size required to achieve a statistical power of 1 − *β* = 0.95 for mixed intra‐ and inter‐subject analyses was 52 participants.

## 3 Results

**FIGURE 1:**
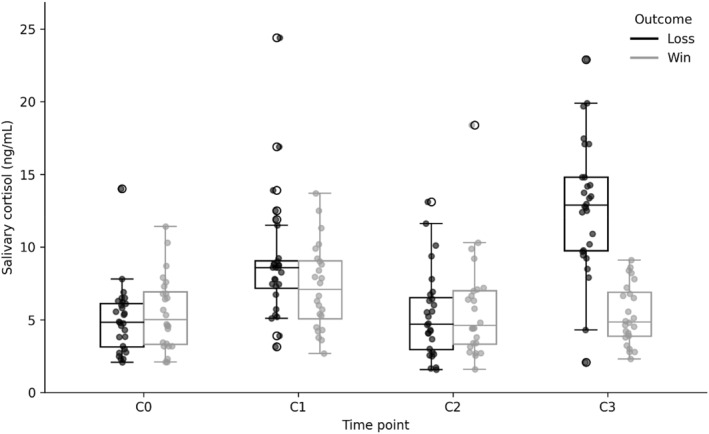
Salivary cortisol concentrations across the competition day according to match outcome. Box‐and‐whisker plots represent the median, interquartile range, and range of salivary cortisol concentrations measured at awakening (C0), 30 min after awakening (C1), pre‐competition (C2), and post‐competition (C3) in players who won or lost the match. Individual data points are shown to illustrate within‐group variability. Cortisol values are expressed in ng/mL.

A two‐way repeated‐measures ANOVA revealed significant within‐subject changes in cortisol concentrations across the competition day (main effect of Time: F(3,197) = 19.25, *p* < 0.001, ges = 0.23). In addition, a significant main effect of match outcome was observed (F(1,197) = 10.19, *p* = 0.002, ges = 0.05), indicating overall differences in cortisol levels between winners and losers. Of note, a relationship between time and outcome was observed (F(3,197) = 11.74, *p* < 0.001, ges = 0.15), suggesting that the temporal pattern of cortisol concentrations differed according to eventual match outcome (Figure 1).

**FIGURE 2:**
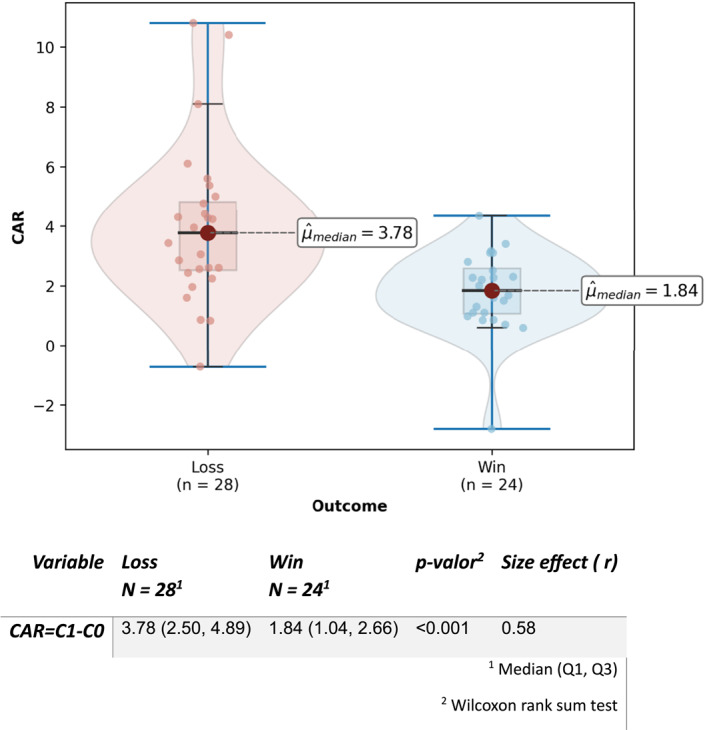
Cortisol awakening response (CAR) by match outcome. Violin plots illustrate the distribution of CAR values (C1–C0) in players who lost (loss) and won (win) the match. Central boxes represent the interquartile range (IQR), horizontal lines indicate the median, and points represent individual observations. CAR was significantly higher in the loss group compared to the Win group (Mann–Whitney *U* = 532, *p* < 0.001), with a large effect size (rank‐biserial *r* = 0.58, 95% CI [0.34, 0.76]).

To characterise and quantify this interaction, logarithmically transformed cortisol concentrations were fitted with linear mixed‐effects models. Table 1 shows that cortisol levels at awakening were similar between winners and losers. However, the Outcome × Time interaction indicated that winners exhibited moderate increases in cortisol 30 min after waking (C1) and decreases upon winning their matches (C3). Figure 2 shows significant differences in CAR according to match outcome, with higher values observed in players who lost than in those who won (median [IQR]: 3.78 [2.50–4.89] vs. 1.84 [1.04–2.66], respectively; Mann–Whitney *U* = 532, *p* < 0.001; rank‐biserial *r* = 0.58; 95% CI [0.34, 0.76]).

**TABLE 1:** Linear mixed‐effects model predicting log‐transformed cortisol concentrations fixed effects estimates (95% CI). Reference categories: Loss, C0 (awakening), forward.

Predictor	*β* (95% CI)
Baseline	
Intercept	1.717*** [1.482, 1.953]
Time effects (loss)	
C1 (+30 min)	0.591*** [0.403, 0.778]
C2 (pre‐competition)	0.007 [−0.180, 0.194]
C3 (post‐competition)	0.947*** [0.760, 1.134]
Outcome (win vs. loss)	
Win	0.100 [−0.156, 0.357]
Outcome × time interactions	
Win × C1	−0.283* [−0.559, −0.008]
Win × C2	−0.005 [−0.281, 0.270]
Win × C3	−0.936*** [−1.211, −0.661]
Playing position (vs. forward)	
Centre	−0.244^a^ [−0.511, 0.023]
Defense	−0.316* [−0.560, −0.071]
Goalkeeper	−0.220 [−0.617, 0.176]

*Note:* Random effects: *σ* ^2^_subject = 0.303; *σ* ^2^_residual = 0.355. Linear mixed‐effects model predicting log‐transformed salivary cortisol concentrations across the competition day. Fixed‐effects estimates (*β*) and 95% confidence intervals (CI) are shown. Reference categories were Loss for match outcome, C0 (awakening) for time, and forward for playing position. Positive coefficients indicate higher cortisol concentrations relative to the reference category. Significant outcome × time interaction terms indicate differential cortisol responses between winners and losers at specific time points. Random effects represent between‐subject variability (athlete‐level intercept) and residual variance.

^a^ *p* < 0.10.

^*^ *p* < 0.05.

**p < 0.01.

^***^ *p* < 0.001.

The CAR showed significant changes dependent on match outcomes; higher concentrations in losers and more attenuated changes in winners (median [IQR]: 3.78 [2.50–4.89] vs. 1.84 [1.04–2.66], respectively; Mann‐Whitney *U* = 532, *p* < 0.001). The effect size indicated moderate to large differences between groups (rank‐biserial *r* = 0.58; 95% CI [0.34; 0.76]).

Exploratory analyses indicated that overall cortisol variability across the competition day differed by playing position (F(3,197) = 5.12, *p* = 0.002, ges = 0.07). Specifically, defensive players exhibited lower overall cortisol concentrations than forwards in the mixed‐model analysis. However, when cortisol responses were examined at individual sampling time points or specifically for CAR values, the differences between playing positions did not reach statistical significance.

Table 2 shows the relationships between hormonal, psychological, and affective variables. Spearman's rank correlation test indicated a psychophysiological response pattern in cortisol production across the competition day; this pattern was associated with psychological and affective states at different times. Cortisol concentrations measured upon waking (C0), 30 min after waking (C1), and before competition (C2) showed moderate to strong intercorrelations (*ρ* = 0.45–0.75, *p* < 0.01). Higher CAR values were statistically associated with greater perceived difficulty and anxiety‐related variables.

**TABLE 2:** Correlation matrix among the measures taken after awakening, pre‐competitive, and post‐competitive moments.

		1	2	3	4	5	6	7	8	9	10	11	12	13	14	15	16	17	18	19	20
1	*C*_0_ (ng/mL)	1	***0.75***	***0.49***	0.19	0.047	−0.12	−0.08	−0.05	0.17	0.01	0.09	0.07	0.05	−0.16	−0.13	0.03	−0.12	0.04	−0.05	−0.06
2	*C*_1_ (ng/mL)		1	***0.45***	***0.40***	***0.623***	0.21	0.13	−0.21	0.14	0.02	0.12	0.27	−0.08	**−0.28**	−0.24	−0.05	0.04	0.03	0.08	−0.13
3	*C*_2_ (ng/mL)			1	0.25	0.072	0.08	0.09	0.05	0.15	0.25	0.15	0.08	0.27	0.01	0.05	0.14	−0.02	0.19	−0.09	0.02
4	*C*_3_ (ng/mL)				1	***0.443***	0.27	**0.34**	−0.01	0.08	0.06	0.14	**0.34**	**0.28**	0.03	−0.04	0.07	0.19	0.08	**0.*46***	0.16
5	CAR					1	***0.39***	0.24	−0.19	0.05	**0.30**	−0.13	0.02	0.05	0.06	**0.30**	−0.21	−0.23	−0.22	−0.17	0.03
6	C.A.						1	***0.37***	−0.21	0.21	0.12	0.24	**0.31**	−0.27	−0.05	0.14	0.11	0.03	−0.09	0.28	−0.15
7	S.A.							1	0.14	0.24	0.16	0.19	0. ***73***	0.15	0.26	−0.13	0.22	−0.04	0.**32**	0. ***57***	0.**34**
8	S.C.								1	0.16	**0.*39***	−0.27	0.04	0.12	***0.42***	0.12	0.01	**−0.28**	***0***. ***47***	−0.03	***0.45***
9	Today is a hard day									1	0.11	0.15	0.24	−0.13	***0.41***	0.16	**0.29**	0.02	0.21	0.13	0.14
10	I am vigorous										1	−0.11	−0.08	0.04	**0.32**	**0.35**	0.22	−0.**35**	**0.35**	0.02	0.**33**
11	Anxiety											1	0.22	0.08	−0.07	−0.09	0.12	0.12	−0.26	0.12	−0.14
12	Nervousness												1	0.07	0.19	−0.14	0.17	0.03	0.**32**	0. ***49***	0.17
13	Inspire my teammates													1	0.21	−0.03	***0.39***	0.21	0.05	0.27	0.**29**
14	Sacrificing myself														1	**0.*38***	***0.42***	−0.10	0.27	**0.27**	**0.*40***
15	Compete with passion															1	0.20	−0.05	0.04	−0.06	0.33
16	My words help the team																1	−0.02	0.08	0.24	***0.43***
17	Defeat is failure																	1	−0.27	−0.07	−0.19
18	Affective valence (S.A.M.)																		1	0.06	***0***. ***41***
19	Arousal (S.A.M.)																			1	0.13
20	Dominance (S.A.M.)																				1

*Note:* Exploratory correlations (Spearman's rho) are shown between cortisol concentrations measured at awakening (C0), 30 min after awakening (C1), pre‐competition (C2), post‐competition (C3), CAR = Cortisol Awakening response, and self‐reported psychological and affective measures, including competitive anxiety dimensions (CSAI‐2R), perceived arousal and vigour, and affective dimensions assessed using the Self‐Assessment Manikin (SAM). Bold values indicate statistically significant correlations (*p* < 0.05), and bold italic values indicate *p* < 0.01. These correlations are intended to describe the associative structure among hormonal and psychophysiological variables and do not imply causal relationships. In bold = *p* < 0.05; in bold and cursive = *p* < 0.01.

Abbreviations: *C*_0_ = salivary cortisol just awakening; *C*_1_ = salivary cortisol 30 min after awakening; *C*_2_ = salivary pre‐competitive cortisol; *C*_4_ = salivary post‐competitive cortisol; C.A. = cognitive anxiety; S.A. = somatic anxiety; S.A.M. = self‐assessment manikin; S.C. = self‐confidence.

CAR demonstrated moderate, direct associations with cognitive anxiety (*ρ* = 0.39, *p* < 0.01), somatic anxiety (*ρ* = 0.24, *p* < 0.05), and the subjective perception that ‘Today is a difficult day’ (*ρ* = 0.30, *p* < 0.05). CAR was also strongly correlated with cortisol measured 30 min after waking (C1; *ρ* = 0.62, *p* < 0.01). These findings suggest that an anticipatory endocrine response upon waking is linked to perceptions of heightened competitive demands and anxiety. In contrast, post‐competitive cortisol concentrations (C3) exhibited consistent relationships with psychological and affective variables. Elevated C3 scores were correlated with somatic anxiety (*ρ* = 0.34, *p* < 0.05), perceived nervousness (*ρ* = 0.34, *p* < 0.05), arousal as measured by the S.A.M. test (*ρ* = 0.46, *p* < 0.01), and team‐directed emotion, specifically ‘Inspiring my teammates’ (*ρ* = 0.28, *p* < 0.05). Post‐competitive cortisol responses were associated with higher scores in arousal‐related and evaluative self‐report measures.

The CSAI‐2R factors revealed associations between cognitive anxiety and somatic anxiety (*ρ* = 0.37, *p* < 0.01), as well as with perceived nervousness (*ρ* = 0.37, *p* < 0.01). Additionally, the self‐confidence factor was related to vigour, self‐sacrificing behaviour, and affective valence as measured by the S.A.M. test. In contrast, self‐reports of pre‐competitive perception and motivation demonstrated specific correlations but did not exhibit a direct or strong association with C concentrations. Overall, the correlational structure suggests partial associations between endocrine, anxiety‐related, and affective variables across the competition day, although causal or mechanistic interpretations cannot be inferred from the present design.

The within‐subject response of C across the four measured time points was moderate to low (ICC (3,1) = 0.37; 95% CI [0.22, 0.52]). The 37% of the variance was modulated by between‐subject differences, while the remaining 63% exhibited within‐subject fluctuations. These results are consistent with the dynamics of C, which are characterised by within‐subject fluctuations across the competition day. These findings justified the combined use of repeated measures and mixed‐effects models to analyse within‐subject hormonal fluctuations.

Finally, competitive experience was not associated with match outcome (*r* = 0.20, *p* = 0.157). Regarding the CAR, only a very weak negative association emerged in the non‐parametric analysis; the Pearson correlation was non‐significant (*r* = −0.18, *p* = 0.193). Importantly, competitive experience did not influence basal cortisol levels, although more experienced players appeared to slightly attenuate the CAR slope.

## 4 Discussion

The present study examined cortisol dynamics across an official competition day in elite female field hockey players competing in a high‐level national championship. The main findings were that: (i) cortisol responses showed substantial inter‐ and intra‐individual variability across the competition day; (ii) players from losing teams exhibited higher cortisol awakening response (CAR) values than winners; (iii) CAR showed partial associations with anxiety‐related and affective variables; and (iv) defeated players displayed higher post‐competition cortisol concentrations than winners. Taken together, these findings suggest that CAR may reflect a context‐sensitive anticipatory endocrine activation associated with competitive demands rather than a direct or deterministic marker of emotional distress or competitive performance.

Previous research has consistently shown that cortisol concentrations tend to increase before competition across a wide range of sports, including swimming, rowing, judo, soccer, volleyball, dance, and triathlon (Balthazar et al. 2012; Doan et al. 2007; Filaire et al. 2001, Filaire et al. 2007, 2009; Kim et al. 2010; Rohleder et al. 2007; van der Meij et al. 2012). These anticipatory endocrine responses have generally been interpreted as associated with cognitive, emotional, and physiological demands in competitive environments rather than with physical exertion alone (Dickerson and Kemeny 2004; Lupien et al. 2009). In this context, the present findings extend previous sport psychophysiology research by showing that distinct cortisol trajectories may emerge between athletes from winning and losing teams during official competition, even when subjective anxiety and affective responses show only partial correspondence with endocrine activity.

Importantly, the present findings should not be interpreted as evidence that CAR determines competitive performance or directly regulates emotional states. CAR was measured before the match outcome was known; therefore, the observed differences should be interpreted as associations with the eventual competitive outcome rather than as outcome‐dependent endocrine regulation. Similarly, although CAR showed moderate associations with cognitive anxiety, somatic anxiety, and arousal‐related variables, these findings indicate only partial coupling between endocrine and subjective responses. This interpretation is consistent with contemporary evidence suggesting that CAR is associated with anticipated demands and psychophysiological preparation rather than functioning as a simple surrogate marker of emotional stress intensity (Clow et al. 2010; Fries et al. 2009; Stalder et al. 2016, 2025).

The lower CAR values observed in winning players may reflect differences in anticipatory psychophysiological activation prior to competition. Previous meta‐analytic evidence indicates that anticipatory cortisol responses vary according to competitive level, sport type, sex, and situational demands (van Paridon et al. 2017). Elite and international‐level athletes frequently display attenuated pre‐competition cortisol responses compared with lower‐level competitors, potentially reflecting habituation to repeated exposure to high‐pressure environments or differences in anticipatory appraisal processes (van Paridon et al. 2017; MacDonald and Wetherell 2019). In the present sample, players from winning teams were slightly older and more experienced, although these differences did not reach statistical significance. Therefore, maturational and experiential factors may plausibly contribute to between‐athlete variability in CAR responses and should be examined more directly in future longitudinal studies.

The interpretation of CAR as a context‐sensitive anticipatory response is also consistent with broader psychoneuroendocrine models proposing that the early morning cortisol increase may be linked to preparation for expected daily demands (Fries et al. 2009). Experimental and naturalistic studies have shown that CAR magnitude tends to increase before anticipated stressors such as examinations, demanding workdays, military training, and socially evaluative situations (Brant et al. 2010; Elder et al. 2018; Kunz‐Ebrecht et al. 2004; Wetherell et al. 2015). Within this framework, the higher CAR values observed in defeated players may reflect differences in anticipatory activation, perceived demands, or competition‐related appraisal processes before the match. However, because the present design was observational and correlational, these interpretations remain tentative and should not be understood as evidence of adaptive or maladaptive endocrine regulation.

The associations observed between CAR and anxiety‐related variables also require cautious interpretation. Previous studies examining relationships between cortisol and competitive anxiety have produced heterogeneous findings, with some reporting positive associations between endocrine activation and somatic or cognitive anxiety and others observing weak or inconsistent relationships (Filaire et al. 2007; Het et al. 2012; Lautenbach et al. 2015; Pineda‐Espejel et al. 2020). In the present study, CAR showed clearer associations with cognitive‐evaluative and arousal‐related variables than with global affective ratings. This pattern may suggest that anticipatory cortisol dynamics are linked more closely to competition‐related appraisal and attentional engagement processes than to conscious emotional distress alone. Nevertheless, because endocrine and psychological variables were assessed simultaneously and correlationally, no causal directionality can be inferred. Consequently, the present findings should not be interpreted as indicating that CAR ‘causes’ anxiety or that anxiety directly drives endocrine activation.

The cortisol awakening response (CAR) in elite female athletes during competition remains poorly understood, despite growing interest in anticipatory endocrine responses and known sex‐related differences. Female athletes remain underrepresented in psychophysiological research, and factors such as ovarian hormones, menstrual cycles, and stress may influence CAR and basal cortisol secretion. Studying CAR in this population could reveal important aspects of endocrine regulation and help address a research gap in women's sports science (Stalder et al. 2025).

An additional finding of the present study was that defeated players displayed higher post‐competition cortisol concentrations than winners. Similar post‐competition cortisol elevations following unsuccessful outcomes have previously been reported in judo, soccer, and other competitive contexts (Doan et al. 2007; Edwards and Kurlander 2010; Oliveira et al. 2009; Reynoso‐Sánchez et al. 2017). Sustained post‐event cortisol activation after defeat may reflect prolonged psychophysiological activation associated with competition outcome, cognitive appraisal, emotional processing, or recovery demands. However, the mechanisms underlying these responses cannot be determined from the present data because the study did not include direct measures of physical load, rumination, coping strategies, emotional regulation, or post‐match recovery processes. Therefore, higher post‐competition cortisol concentrations in defeated athletes should be interpreted cautiously and not attributed to any single psychological or physiological mechanism.

An important aspect of the present findings is the substantial inter‐ and intra‐individual variability observed in cortisol responses across the competition day. The intraclass correlation analyses indicated that a substantial proportion of cortisol variance occurred within rather than between athletes, supporting the interpretation that endocrine responses during competition are highly dynamic and context dependent. This observation aligns with previous sport psychophysiology literature emphasising the importance of within‐athlete monitoring approaches when interpreting endocrine responses in elite sport settings (Anderson and Wideman 2017; Crewther et al. 2024, 2025). Rather than assuming fixed hormonal profiles, these findings support the idea that cortisol responses fluctuate according to situational demands, anticipatory processes, and competition‐related contexts.

Several methodological limitations should be acknowledged. First, CAR assessment was based on a two‐sample protocol and did not include objective compliance verification procedures such as actigraphy or time‐stamped sampling devices. Although abbreviated CAR protocols have previously demonstrated sensitivity to competition‐related endocrine changes in ecologically valid elite‐sport settings (MacDonald and Wetherell 2019), they do not capture the full temporal dynamics of cortisol secretion recommended in current CAR consensus guidelines (Stalder et al. 2016). Second, menstrual cycle phase, sleep quality, awakening time variability, and prior‐day physical and psychological load were not controlled, all of which may influence CAR magnitude and cortisol regulation (Stalder et al. 2016). Third, the correlational analyses were exploratory and should therefore be interpreted cautiously. Finally, because the study was conducted during a single official competition day, the findings should not be generalised to long‐term endocrine adaptation or interpreted within broader models of chronic stress.

Despite these limitations, the present study provides ecologically valid evidence regarding anticipatory cortisol dynamics in elite female athletes competing under official high‐stakes conditions. The findings support the interpretation that CAR may reflect a context‐sensitive anticipatory response associated with forthcoming competitive demands and eventual outcome, while remaining only partially associated with subjective anxiety and affective states. Importantly, the results also highlight the complexity of psychophysiological responses during elite competition and suggest that endocrine activity and subjective emotional experience should not be assumed to operate in a simple one‐to‐one manner. Future research should incorporate repeated competition and non‐competition measurements, objective compliance verification procedures, menstrual cycle monitoring, and integrated psychophysiological workload assessments to better characterise the role of CAR in elite sport contexts and clarify how anticipatory endocrine responses interact with cognitive, affective, and performance‐related processes over time.

## 5 Conclusions

Elite female field hockey players showed marked variability in cortisol dynamics across the competition day. Higher cortisol awakening response (CAR) values and post‐competition cortisol concentrations were observed in players from teams that eventually lost, while CAR also showed partial associations with anxiety‐related and affective variables. These findings suggest that anticipatory cortisol responses may reflect psychophysiological preparation for competition under high‐level ecological conditions rather than a direct marker of performance or emotional stress. However, given the observational design and single‐competition‐day assessment, the results should be interpreted cautiously and confirmed through longitudinal studies incorporating additional psychophysiological and contextual measures.

### 5.1 Study Limitations

Several limitations should be considered when interpreting the present findings. First, the sample size was relatively modest, which may have limited statistical power for some subgroup and interaction analyses, particularly those involving playing position and match outcome comparisons. In addition, the study was conducted during a single official competition day in a specific elite female sport context, which may limit the generalisability of the findings to other sports, competitive levels, or competition formats.

Second, the observational and cross‐sectional nature of the study prevents causal inference. Although CAR was associated with eventual competitive outcome and several anxiety‐related variables, these associations should not be interpreted as evidence that cortisol responses determine performance or directly regulate emotional states. Rather, the present findings reflect associations observed under ecologically valid competitive conditions.

Third, several methodological factors known to influence cortisol dynamics were not controlled. Menstrual cycle phase, sleep quality, awakening variability, prior‐day physical and psychological load, and anticipatory cognitive states immediately upon awakening were not assessed. In addition, CAR was evaluated using a two‐sample protocol without objective adherence verification procedures such as actigraphy or time‐stamped sampling devices. Although abbreviated CAR protocols have previously been used in elite sport settings with acceptable sensitivity (MacDonald and Wetherell 2019), the present approach does not capture the full temporal dynamics of cortisol secretion, as recommended in current consensus guidelines (Stalder et al. 2016). Consequently, the present findings should be interpreted specifically in relation to the initial post‐awakening increase in cortisol rather than as a comprehensive characterisation of the full CAR profile.

Another limitation concerns the psychological assessment procedures. Anxiety and affective states were evaluated using self‐report questionnaires, which may be influenced by social desirability, emotional awareness, motivational factors, or response biases commonly observed in high‐performance sport environments. Furthermore, psychological variables and endocrine responses were assessed correlationally and at partially overlapping time points, preventing conclusions regarding temporal or causal directionality between subjective experience and hormonal activity. Therefore, the observed associations between CAR and anxiety‐related variables should be interpreted as evidence of partial psychophysiological coupling rather than direct psychobiological causation.

Finally, the study did not include objective measures of in‐match physical load, cognitive load, autonomic activation, or post‐competition recovery processes. Consequently, the mechanisms underlying the higher post‐competition cortisol concentrations observed in defeated players cannot be determined from the present data. These responses may reflect a combination of physiological activation, competition‐related appraisal processes, emotional processing, and recovery demands rather than any single psychological mechanism. Future studies should incorporate longitudinal competition monitoring, objective compliance verification procedures, menstrual cycle assessment, sleep and workload monitoring, and multimodal psychophysiological measures to better characterise anticipatory cortisol dynamics in elite athletes under real‐world competitive conditions.

## Funding

This work was supported by Universidad Internacional de La Rioja under grant number [COD‐FITPRO‐B0036/2016]. The funder had no role in study design, data collection and analysis, decision to publish, or preparation of the manuscript.

## Conflicts of Interest

The authors declare no conflicts of interest.

## Supporting information


Supporting Information S1

**Table S1:** Participant characteristics and descriptive statistics by match outcome values are reported as median (standard deviation [SD], interquartile range [IQR]).

## Data Availability

The data that supports the findings of this study are available in the supplementary material of this article.
