# Supplementary material for: Anticipatory Cortisol Regulation During Competition in Elite Female Field Hockey Players
Source: Stress Health. 2026 Jul 5;42(4):e70199. doi: 10.1002/smi.70199 (PMC13333907; doi:10.1002/smi.70199)
Supplement: Supplementary file 2 — Table S1: Participant characteristics and descriptive statistics by match outcome values are reported as median (standard deviation [SD], interquartile range [IQR]). [file SMI-42-e70199-s002.docx]

**Supplement material.**

**Table S1. Participant characteristics and descriptive statistics by match outcome**

**Values are reported as Median (standard deviation [SD], interquartile range [IQR])**

**Participant characteristics**

| **Variable** | **Loss (n = 28)** | **Win (n = 24)** |
| --- | --- | --- |
| **Age (years)** | 22.0 (3.37; 3.0) | 26.0 (3.97; 5.5) |
| **Competitive experience (years)** | 16.0 (4.14; 4.0) | 17.5 (4.84; 7.5) |

**Hormonal measures**

| **Variable** | **Loss** | **Win** |
| --- | --- | --- |
| **C0 (ng/ml)** | 4.84 (2.39; 2.97) | 5.01 (2.54; 3.61) |
| **C1 (ng/ml)** | 8.59 (4.22; 1.89) | 7.10 (2.93; 3.97) |
| **C2 (ng/ml)** | 4.70 (2.96; 3.57) | 4.62 (3.61; 3.67) |
| **C3 (ng/ml)** | 12.89 (4.54; 5.04) | 4.85 (2.06; 3.02) |
| **CAR (C1–C0)** | 3.78 (2.59; 2.30) | 1.84 (1.39; 1.51) |

**Psychological and perceptual variables**

| **Variable** | **Loss** | **Win** |
| --- | --- | --- |
| Cognitive anxiety (CSAI-2R) | 29.0 (4.34; 5.25) | 26.0 (3.92; 5.00) |
| Somatic anxiety (CSAI-2R) | 24.0 (4.87; 7.50) | 22.0 (4.29; 7.00) |
| Self-confidence (CSAI-2R) | 31.0 (5.02; 8.25) | 32.0 (4.61; 7.00) |
| Today is a hard day | 7.0 (1.12; 1.00) | 6.0 (0.93; 1.00) |
| I am vigorous | 7.0 (1.33; 2.00) | 6.0 (1.29; 2.00) |
| Perceived anxiety | 6.0 (1.43; 2.00) | 5.0 (1.29; 2.00) |
| Perceived nervousness | 6.0 (1.37; 2.00) | 5.0 (1.33; 2.00) |
| Inspire my teammates | 7.0 (1.43; 1.00) | 6.0 (1.07; 1.00) |
| Sacrificing myself | 9.0 (0.74; 1.00) | 9.0 (0.83; 1.00) |
| Compete with passion | 8.0 (1.17; 2.00) | 8.0 (0.70; 1.00) |
| My words help the team | 7.0 (1.40; 1.25) | 6.5 (1.36; 1.25) |
| Defeat is failure | 6.0 (1.69; 2.00) | 5.0 (1.22; 1.00) |
| Affective valence (SAM) | 7.0 (1.10; 1.00) | 7.0 (1.01; 1.00) |
| Arousal (SAM) | 7.0 (1.37; 2.00) | 6.0 (1.35; 1.00) |
| Dominance (SAM) | 7.0 (1.47; 2.00) | 7.0 (1.10; 1.00) |

**📝 Footnote (journal-ready)**

*C0 = cortisol upon awakening; C1 = cortisol 30 min after awakening; C2 = pre-competitive cortisol; C3 = post-competitive cortisol; CAR = cortisol awakening response (C1–C0). CSAI-2R = Competitive State Anxiety Inventory–2 Revised; SAM = Self-Assessment Manikin. Perceptual and motivational variables correspond to single-item self-reports. Median, standard deviation (SD), and interquartile range (IQR) are reported to allow comparison across distributional assumptions.*
